# Maternal Heat Stress Alters Expression of Genes Associated with Nutrient Transport Activity and Metabolism in Female Placentae from Mid-Gestating Pigs

**DOI:** 10.3390/ijms22084147

**Published:** 2021-04-16

**Authors:** Weicheng Zhao, Fan Liu, Christina D. Marth, Mark P. Green, Hieu H. Le, Brian J. Leury, Alan W. Bell, Frank R. Dunshea, Jeremy J. Cottrell

**Affiliations:** 1 Faculty of Veterinary and Agricultural Sciences, University of Melbourne, Parkville, VIC 3010, Australia; christina.marth@unimelb.edu.au (C.D.M.); huul1@student.unimelb.edu.au (H.H.L.); brianjl@unimelb.edu.au (B.J.L.); fdunshea@unimelb.edu.au (F.R.D.); 2 Rivalea Australia Pty Ltd., Corowa, NSW 2646, Australia; fliu@rivalea.com.au; 3 Faculty of Science, University of Melbourne, Parkville, VIC 3010, Australia; mark.green@unimelb.edu.au; 4 Department of Animal Science, Cornell University, Ithaca, NY 14853-4801, USA; awb6@cornell.edu; 5 Faculty of Biological Sciences, The University of Leeds, Leeds LS2 9JT, UK

**Keywords:** placental insufficiency, heat stress, gestation, RNA-seq, placenta, transport activity, pigs

## Abstract

Placental insufficiency is a known consequence of maternal heat stress during gestation in farm animals. The molecular regulation of placentae during the stress response is little known in pigs. This study aims to identify differential gene expression in pig placentae caused by maternal heat exposure during early to mid-gestation. RNA sequencing (RNA-seq) was performed on female placental samples from pregnant pigs exposed to thermoneutral control (CON; constant 20 °C; *n* = 5) or cyclic heat stress (HS; cyclic 28 to 33 °C; *n* = 5) conditions between d40 and d60 of gestation. On d60 of gestation, placental efficiency (fetal/placental weight) was decreased (*p* = 0.023) by maternal HS. A total of 169 genes were differentially expressed (FDR ≤ 0.1) between CON and HS placentae of female fetuses, of which 35 genes were upregulated and 134 genes were downregulated by maternal HS. The current data revealed transport activity (FDR = 0.027), glycoprotein biosynthetic process (FDR = 0.044), and carbohydrate metabolic process (FDR = 0.049) among the terms enriched by the downregulated genes (HS vs. CON). In addition, solute carrier (SLC)-mediated transmembrane transport (FDR = 0.008) and glycosaminoglycan biosynthesis (FDR = 0.027), which modulates placental stroma synthesis, were identified among the pathways enriched by the downregulated genes. These findings provide evidence that heat-stress induced placental inefficiency may be underpinned by altered expression of genes associated with placental nutrient transport capacity and metabolism. A further understanding of the molecular mechanism contributes to the identification of placental gene signatures of summer infertility in pigs.

## 1. Introduction

Placentae are the primary interface between gestating dams and fetuses, supporting fetal development. Therefore, factors that influence placental function may affect prenatal development of fetuses and reproductive efficiency of farm animals. In litter bearing animals, such as the pig, placental efficiency (fetal/placental weight) is a major determinant for prenatal fetal survival, as there is competition between fetuses for maternal nutrients and limited intrauterine space [1,2]. Compromised placental efficiency implies an insufficient capacity at a given unit mass of the placenta to support fetal growth. One factor potentially contributing to impaired placental function is maternal heat stress (HS) during gestation, as this can contribute to intrauterine growth restriction (IUGR) and cause implications for birthweight and postnatal development [3,4]. A recent study showed that piglets born from sows mated in summer had lower average birthweights compared to their counterparts from autumn-mated sows [5]. There is growing evidence that pig progeny experiencing prenatal heat stress have an altered postnatal phenotype, including increased carcass fatness and impaired reproductive performances (as reviewed by Johnson et al. [6]). In pregnant sheep, high ambient temperatures result in placental insufficiency and IUGR [3,7,8], possibly associated with reductions in uterine blood flow, as a consequence of heat adaptation of animals [9]. Hence, reduced uterine blood flow may decrease placental transport capacity for nutrients and oxygen between maternal and fetal circulations [3,10,11,12].

Factors affecting pig placental efficiency are mostly associated with placental morphology and nutrient transport capacity [13]. Therefore, it is speculated that the impact of maternal heat stress on placental efficiency may be underpinned by changes in the expression of relevant genes. In particular, recent studies have revealed the role of placental nutrient transport capacity, especially the gene expression of transmembrane transporters, on placental efficiency in pigs [13,14]. We have previously demonstrated that exposing pregnant pigs to elevated temperatures during early to mid-gestation decreases placental efficiency, as reflected by a compensatory enlargement of the placenta without an accompanying change in fetal weights [15]. These findings emphasise the susceptibility of pig placentae to maternal thermal stress, especially during the critical time window of placental development [16]. However, the molecular and cellular responses resulting in identified placental insufficiency and morphological adaptations remain unknown. In the present study, we proposed to test the hypothesis that maternal heat stress during early to mid-gestation (d40 to d60) affects the global expression of genes related to placental nutrient transport capacity and metabolism.

## 2. Results

### 2.1. Fetal and Placental Morphometry

Data analysed herein represent a subset of samples obtained from a larger study reported elsewhere [15]. Placental and fetal morphology data are presented in Table 1. On d60 of gestation, individual fetal weights tended to be decreased (*p* = 0.08) with maternal heat treatment, whereas individual placental weights (*p* = 0.053) and placental surface area (*p* = 0.09) tended to be increased with maternal heat treatment. Placental efficiency (fetal/placental weight) was lower (*p* = 0.023) in the HS group compared to the control (CON) group (Table 1).

### 2.2. Differentially Expressed Genes, and Gene Ontology and Pathway Enrichment Analyses

**Figure 1:**
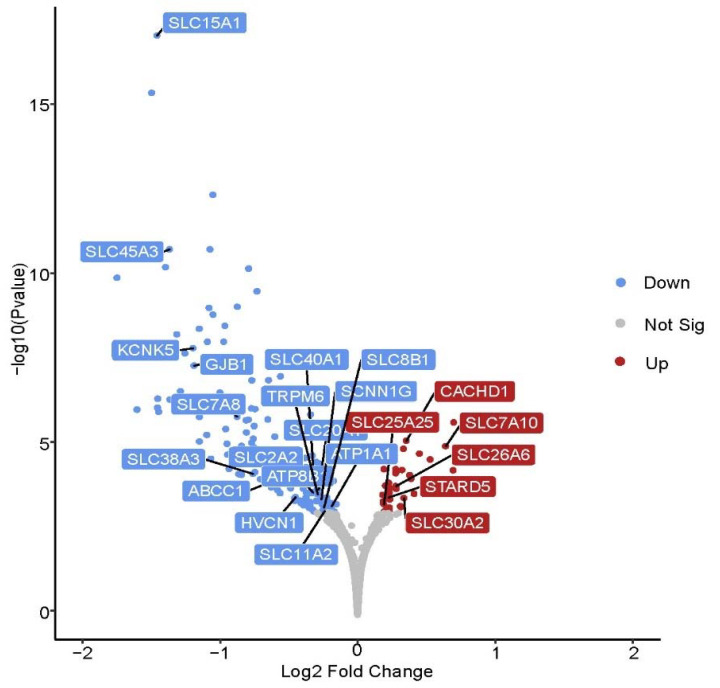
Volcano plot of the differentially expressed genes (DEGs) in female placentae. Blue, red, and grey dots denote downregulated ((FDR (false discovery rate) ≤ 0.1), upregulated (FDR ≤ 0.1), and non-differentially expressed (FDR > 0.1) genes in heat stress placentae (*n* = 5) compared to control placentae (*n* = 5). Gene symbols associated with placental transport activity are labelled.

**Figure 2:**
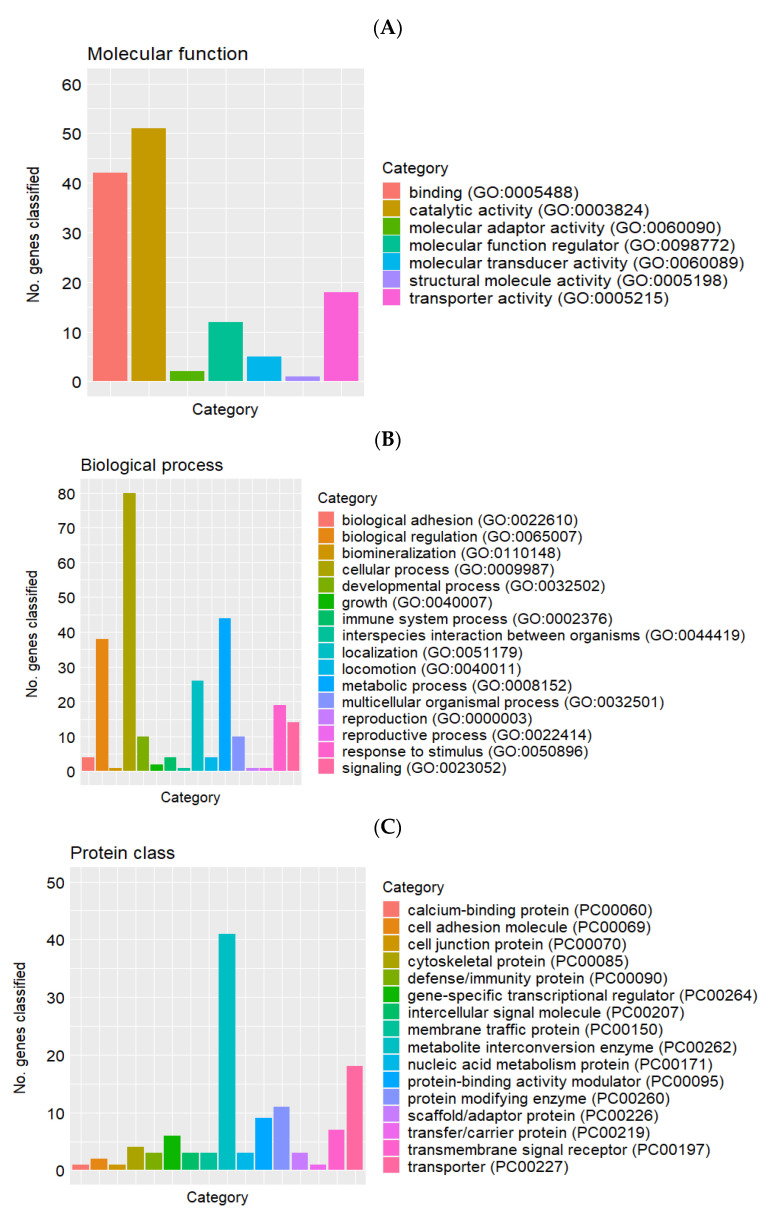
Functional classification of differentially expressed genes in placentae between heat stress (*n* = 5) and control (*n* = 5) groups in the gene ontology (GO) domains of (**A**) Molecular function, (**B**) Biological process and (**C**) Protein class.

A total of 169 genes were differentially expressed (FDR (false discovery rate) ≤ 0.1) in placentae from female fetuses between the CON and HS groups (Figure 1). A subset of the differentially expressed genes (DEGs) is listed in Table 2. It includes the top five most upregulated and downregulated genes by fold change, and genes associated with transport activity, carbohydrate metabolism, glycosaminoglycan biosynthesis, and extracellular vesicles that were a particular focus herein. When compared with the CON group, a total of 35 genes were upregulated, and 134 genes were downregulated by maternal heat stress. Functional classification of these DEGs is presented in Figure 2A–C. In terms of the gene ontology (GO) domain of molecular function (Figure 2A), the DEGs were mostly assigned to catalytic activity (GO:0003824; 51 genes), binding (GO:0005488; 42 genes), and transporter activity (GO:0005215; 18 genes). Regarding the GO domain of biological process (Figure 2B), the DEGs were mostly assigned to cellular process (GO:0009987; 80 genes), metabolic process (GO:0008152; 44 genes), and biological regulation (GO:0065007; 38 genes). Protein classification (Figure 2C) of the DEGs identified metabolite interconversion enzyme (PC00262; 41 genes), transporter (PC00227; 18 genes), and protein modifying enzyme (PC00260; 11 genes) among the protein categories with the most assigned DEGs.

**Figure 3:**
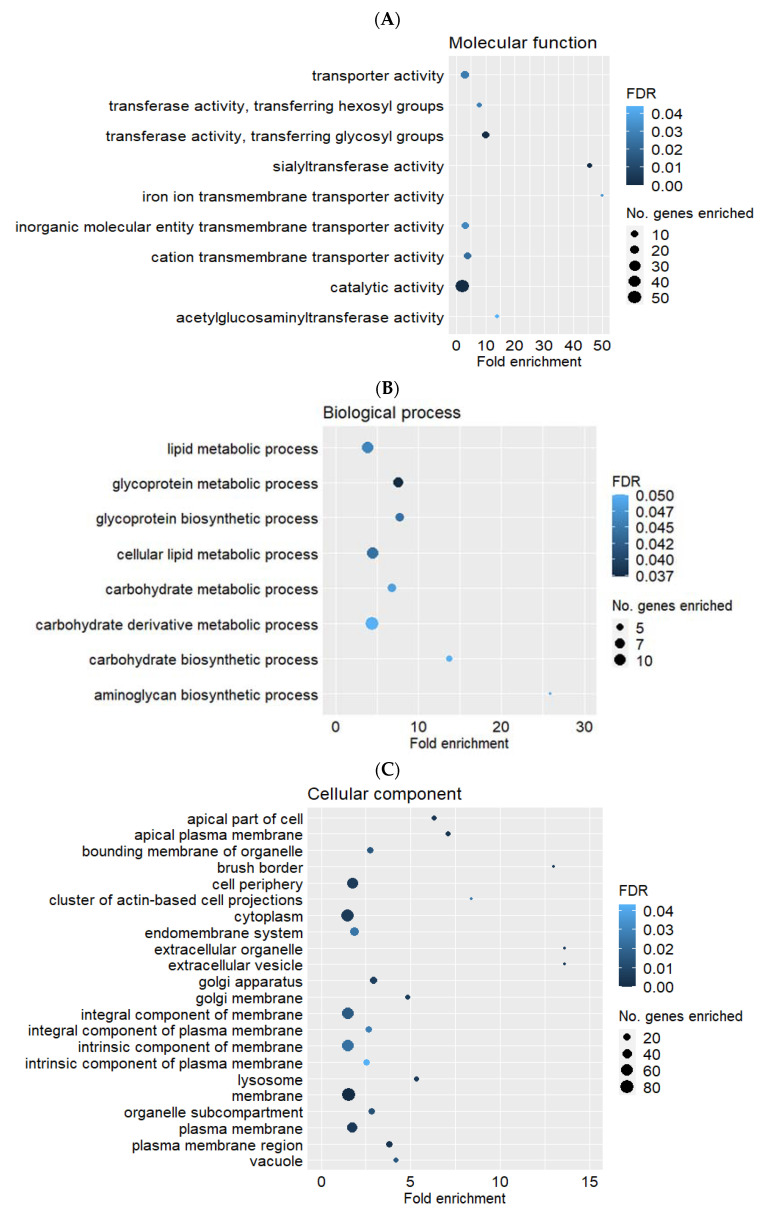
Gene ontology (GO) terms significantly enriched (FDR ≤ 0.05) by the downregulated genes in heat stress placentae (*n* = 5) compared to control placentae (*n* = 5) in the GO domains of (**A**) Molecular function, (**B**) Biological process and (**C**) Cellular component. Fold enrichment: No. genes observed in the gene list/No. genes expected.

The upregulated and downregulated DEGs (HS compared with CON) were analysed separately by the statistical overrepresentation test, and the GO terms significantly enriched by the downregulated genes are depicted in Figure 3A–C. In the GO domain of molecular function (Figure 3A), maternal heat treatment decreased expression of genes associated with transporter activity (GO:0005215; FDR = 0.027), such as cation transmembrane transporter activity (GO:0008324; FDR = 0.023) and inorganic molecular entity transporter activity (GO:0015318; FDR = 0.031); and catalytic activity (GO:0003824; FDR < 0.001), including sialyltransferase activity (GO:0008373; FDR < 0.001). In the GO domain of biological process (Figure 3B), placental metabolic processes, such as aminoglycan biosynthetic process (GO:0006023; FDR = 0.052), carbohydrate metabolic process (GO:0005975; FDR = 0.049), glycoprotein biosynthetic process (GO:0009101; FDR = 0.044) and lipid metabolic process (GO:0006629; FDR = 0.046), were significantly enriched by the downregulated genes (HS compared with CON). In the GO domain of cellular component (Figure 3C), extracellular vesicle (GO:1903561; FDR < 0.001) and brush border (GO:0005903; FDR = 0.014) were the enriched GO terms with the highest fold enrichment by the downregulated genes (HS compared with CON). Despite there being 35 genes upregulated by maternal heat treatment, no GO term was significantly enriched (FDR > 0.05) by the upregulated genes (HS compared with CON). The full list of significantly enriched GO terms with their associated genes, gene ontology ID, FDR, and fold enrichment are listed in Table S1.

A subset of significantly enriched (FDR ≤ 0.05) Reactome and KEGG (Kyoto Encyclopedia of Genes and Genomes) pathways by the downregulated DEGs (HS compared with CON) is listed in Table 3. Placental metabolism, including glycosaminoglycan biosynthesis—keratan sulfate (KEGG:00533; FDR = 0.027) and metabolism of carbohydrates (R-SSC-71387; FDR < 0.001); and solute carrier (SLC)-mediated transmembrane transport (R-SSC-425407; FDR = 0.008) were among the pathways enriched by the downregulated genes (Table 3). The full list of significantly enriched pathways with their associated genes, pathway ID, and FDR are listed in Table S2. No pathway was significantly enriched (FDR > 0.05) by the 35 upregulated genes (HS compared with CON).

### 2.3. Western Blot 

**Figure 4:**
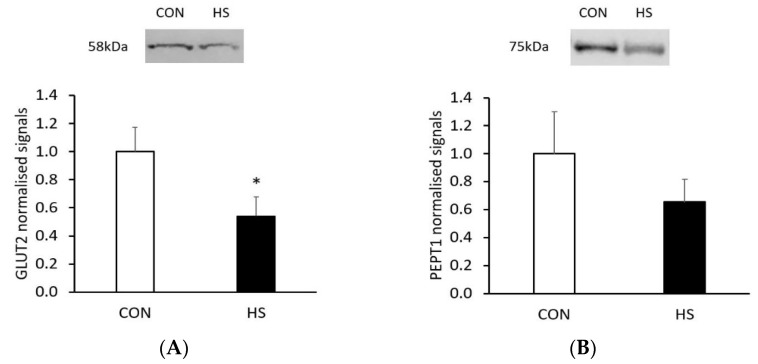
Relative protein expression of glucose transporter 2 (GLUT2; (**A**)) and peptide transporter 1 (PEPT1; (**B**)) in placentae between control (CON, *n* = 5) and heat stress (HS, *n* = 5) groups. Target protein expression was normalised to total protein loaded and expressed as normalised signals (arbitrary units). * *p* < 0.05. Error bars: standard error of the means.

The relative protein expression of glucose transporter 2 (GLUT2) and peptide transporter 1 (PEPT1) encoded from two candidate genes, solute carrier family 2 member 2 (*SLC2A2*) and *SLC15A1*, respectively, was quantified by western blot. Consistent with the gene expression change, relative protein expression of GLUT2 was lower in the HS group compared to the CON group (*p* = 0.036, Figure 4A). However, relative expression of PEPT1 was not significantly different (*p* = 0.18) between placentae from the two groups (Figure 4B). The original blot images for GLUT2 and PEPT1 were provided in Supporting Information File 1.

## 3. Discussion

The findings of this study reinforce our previous observations of placental insufficiency and compensatory adaptation of placentae in response to maternal heat stress during the critical time window of gestation in pigs [15]. The current data also extend the previous findings by revealing molecular mechanisms underpinning the phenotypes identified. The principal findings of this study support our hypothesis, as they clearly showed that maternal heat stress altered the expression of genes associated with placental nutrient transporter activity and metabolism in pregnant pigs. Particularly, the data revealed decreased expression of genes associated with the SLC-mediated transmembrane transport by maternal heat stress. Placental transport of most nutrients is facilitated by transporters located on the placental membranes, hence, altered gene expression of these transporters suggests a changed substrate transport capacity of the placentae [13].

We have identified that maternal heat stress decreased the expression of genes associated with major nutrient transporters, including those for neutral amino acids and peptides. Genes encoding neutral amino acid transporters (*SLC7A8* and *SLC38A3*) are among the downregulated genes that are associated with SLC-mediated transmembrane transport. Gene *SLC7A8* has previously been detected in the pig placenta [13], and the protein product of *SLC7A8* is a sodium-independent system L amino acid exchanger (LAT2) that facilitates transport of large neutral amino acids, including glutamine, leucine, and alanine [17]. Gene *SLC38A3* encodes a sodium-dependent system N amino acid transporter (SNAT3) and mediates transport of glutamine, histidine, and asparagine [18]. Decreased gene expression of those genes may have implications for transplacental amino acid transport, especially of glutamine, which is a critical amino acid for fetal development in pigs [19]. Previous studies have reported reduced placental leucine and threonine fluxes by gestational heat stress in gestating dams in a sheep model of fetal growth restriction [10,11]. In the present study, we also identified gene *SLC15A1*, which encodes the high capacity di/tripeptide transporter (PEPT1), to be among the most downregulated genes by maternal heat stress. Although the gene product of *SLC15A1* is mostly known as a high-capacity peptide transporter located in the intestinal epithelium [20], this gene has previously been detected in trophoblast epithelial cells of the pig placenta, supporting its possible involvement in transplacental peptide transport [13]. However, a significant change in relative protein expression of PEPT1 was not detected in this study, although it was numerically lower in HS placentae compared to CON placentae. The reason for this observation may be that the steady-state protein expression may be subjected to post-transcriptional modifications and protein turnover rates [21]. Nevertheless, these findings highlight the altered expression of genes associated with placental neutral amino acid and peptide transport by maternal heat exposure. However, whether the change in gene expression contributes to a functional change of those transporters and subsequently affects fetal uptake of amino acids needs to be further investigated.

In this study, the GO term of ion transmembrane transporter activity was also enriched by the differentially expressed genes. For example, lower gene expression of ATPase isoforms (*ATP1A1*, *ATP13A3*, *ATP8B1*) were detected in HS placentae compared with CON placentae. The protein product of gene *ATP1A1* is a subfamily of sodium-potassium ATPase, known as the sodium pump, which plays an essential role in maintaining cellular electrochemical gradients and contributes to the net transport of sodium across plasma membranes [22]. Notably, the activity of sodium-potassium ATPase is known to be reduced in human placentae from IUGR pregnancies [23]. In the current study, a lower gene expression of *ATP1A1* is combined with a decreased expression of gene *SCNN1G*, which encodes a subunit of epithelial sodium channel (ENaC) [24]. It has been reported that the ENaC protein is expressed in placentae of mid to late-gestating pigs, mediating the net movement of sodium across placental membranes [25]. Thus, changes in expression of these genes may suggest altered placental cellular ionic homeostasis, especially of the sodium ion, due to maternal thermal stress. Equally in the present study, gene expression of *SLC40A1* and *SLC11A2*, which encode ferroportin and divalent metal transporter-1 (DMT1), respectively [26], were also lower in HS placentae. Ferroportin and DMT1 are two well-known proteins critical for cellular iron transport [27]. Ferroportin mediates iron efflux from the cell, whereas DMT1 acts as an iron importer [26,27]. Although it is known that transplacental iron transport is partially regulated by placental synthesis and secretion of uteroferrin [28], decreased gene expression of ferroportin (*SLC40A1*) and DMT1 (*SLC11A2*) may affect iron fluxes and homeostasis on a cellular level, subsequently affecting transmembrane transport of iron ions.

Pathway enrichment analysis showed that glycosaminoglycan biosynthesis, by which placental stroma is synthesised, was enriched by the downregulated genes. This suggests that placental stroma synthesis is downregulated by maternal heat stress. Placental stroma, together with its embedded epithelial bilayer, are two major structures of pig placentae [13]. In addition, placental stroma is composed of extracellular matrix components, which are biosynthesised by glycosaminoglycans [29]. Therefore, altered gene expression associated with the glycosaminoglycan biosynthesis may have implications for placental stroma morphology. The mechanism by which maternal heat stress affects glycosaminoglycan biosynthesis and placental stroma development remains unknown. One possible explanation is that it is part of a placental compensatory mechanism in an attempt to increase nutrient transport capacity. This can be achieved by enhancing the growth of placental epithelium, as the pig epithelial bilayer develops at the cost of the width of placental stroma [30]. The current finding is in accordance with our previously reported placental microscopy results, showing placentae from heat-stressed gilts had an increased epithelial layer thickness than placentae from the control gilts [15]. Therefore, downregulation of the glycosaminoglycan biosynthesis may instead mean that there is a dilution of the stroma in the HS placenta, contributing to compensatory growth of the epithelial bilayer and the nutrient exchange surface area. Collectively, this adaptative strategy aims to compensate for the heat-stress induced placental insufficiency in pigs.

Furthermore, this study identified that the altered glycosaminoglycan biosynthesis could be associated with downregulated expression of key genes relevant to its upstream metabolic pathways. Notably, the expression of *SLC2A2*, a gene encoding the glucose transporter 2 (GLUT2), was lower in HS placentae compared to CON placentae. Although gene expression of *SLC2A2* has previously been detected in pig placentae [31], the role of GLUT2 in placental glucose transport is poorly understood. Interestingly, it has been suggested that GLUT2 may be involved in transport of glucosamine needed for glycosaminoglycan biosynthesis [32,33]. In this study, the reduction in *SLC2A2* expression in the placenta from the HS group was reinforced by lower relative protein expression of GLUT2, suggesting a change in biological function. We also identified fructose-1,6-bisphosphatase 2 (*FBP2*) and glucose-6-phosphate isomerase (*GPI*) among the downregulated genes. The gene product of *GPI* catalyses isomerisation of glucose 6-phosphate to fructose 6-phosphate in the glycolytic pathway, whereas the gene product of *FBP2* catalyses fructose 1,6-biphosphate to fructose 6-phosphate in the gluconeogenesis pathway [34]. Interestingly, the protein products of *FBP2* and *GPI* are both involved in the synthesis of fructose 6-phosphate. Fructose 6-phosphate, together with glutamine, are important substrates for the synthesis of glycosaminoglycans via the hexosamine biosynthesis pathway in pig placentae [31,35]. In a previous study, Krombeen et al. [36] reported that *FBP2* gene expression was increased in placentae with higher placental efficiency in late gestating pigs. Taken together, the current results demonstrate that maternal heat stress affects the expression of genes associated with placental synthesis of glycosaminoglycans, the primary component of placental stroma, suggesting altered placental metabolism and function.

An interesting finding of this study is that the cellular component GO term extracellular vesicle was enriched, based on five genes (*GPRC5C*, *SLC11A2*, *SCNN1G*, *DEFB1*, and *ANXA1*). Extracellular vesicles, which contain proteins, small RNAs, and lipids, are bilayer membranous complexes released into the extracellular microenvironment [37]. During gestation, especially at the early stages, extracellular vesicles are known to transport biologically active molecules and to act as a vehicle of intracellular communication in uterine luminal fluids [38] and placentae [39], favouring conceptus development. In pig placentae, extracellular vesicles are known mediators of placental endothelial cell proliferation and angiogenesis during early gestation [39]. Although the mechanism by which heat stress affects intracellular communication and signalling via extracellular vesicles remains poorly understood, a recent study has suggested a protective role of extracellular vesicles in combating heat-stress induced oxidative stress in follicular cells [40]. The present data are also in line with a previous study showing that extracellular exosomes, a subset of extracellular vesicles, were enriched by differentially expressed genes between pig placentae with high and low placental efficiency [36]. Collectively, these findings highlight the notion that changes in molecular regulation of extracellular vesicles may be implicated in differences in placental efficiency and heat stress responses.

In terms of future studies, the current study was performed on placentae from female fetuses, due to female placentae having a more pronounced response to maternal heat stress [15], and to limit the impacts of intra-litter variation. It would therefore be of interest to investigate and quantify sexual dimorphic differences in the genes and pathways highlighted in the present study. Based on the fundamental gene ontology and pathways identified in the current study, we predict that many of the reported changes would also respond, although to a lesser extent, to maternal heat stress in placentae of male fetuses. In this study, it remains unclear whether the identified molecular responses and placental insufficiency are directly associated with maternal hyperthermia or indirectly related to maternal heat adaptations, such as a reduction in uterine blood flow in an attempt to increase periphery heat dissipation [9]. Reduced uterine blood flow may affect placental transport efficiency by, at least in part, affecting ion gradients and transport of oxygen. Future studies are required to gain a better understanding of the exact mechanism.

## 4. Conclusions

The findings of this study confirm that maternal heat stress during early to mid-gestation in pigs decreased the expression of genes associated with placental nutrient transport activity, including neutral amino acid, peptide, and cation transmembrane transport. These data highlight that placental insufficiency induced by maternal heat stress may be underpinned by impaired placental nutrient transport capacity. Furthermore, altered expression of genes related to placental metabolism, such as the downregulation of glycosaminoglycan (placental stroma) biosynthesis, provides a mechanism to explain placental compensatory adaptations in an attempt to mitigate against the placental insufficiency in order to sustain fetal development.

## 5. Materials and Methods

### 5.1. Ethical Approval

The animal procedures were reviewed and approved by the Animal Ethics Committee (AEC) of the University of Melbourne, Australia (Ethics Id: 1714365.2, 3 October 2018). The experimental protocols followed the Australian Code for the Care and Use of Animals for Scientific Purposes (8th edition; National Health and Medical Research Council, 2013).

### 5.2. Animals and Experimental Design

Placental samples used for RNA-seq in the present study were obtained from a subset of tissues collected from animals used in a previous experiment [15]. Briefly, ten Large White × Landrace female pigs (gilts; 112 ± 5 kg liveweights, mean ± SD) were selected from a commercial piggery and were artificially inseminated (d0) on a farm (Huntly Piggery, Victoria, Australia). After pregnancy was confirmed at d28 of gestation, the pregnant pigs were transported to the climate-controlled facility at the University of Melbourne. The pigs were individually housed in plastic floor pens (2.2 m × 1.2 m) at constant 20 °C for acclimation. On d40 of gestation, pregnant pigs were either exposed to thermoneutral control (CON; *n* = 5; constant 20 °C) or cyclic heat stress conditions (HS; *n* = 5; 33 °C between 0900 h and 1700 h, and 28 °C between 1700 h and 0900 h) for 3 weeks. The heat treatment mimicked hot summer conditions in Australian pig farms. During the experimental period, pregnant pigs were restrictively fed with a commercially gestating diet at 2.0 kg per day as per commercial procedures. All pigs had equivalent nutrition intakes and had *ad libitum* access to water during the experimental period. Overall, the climate-controlled period was between d40 and d60 of gestation. 

### 5.3. Tissue Collection and Morphometric Analysis 

On d60 of pregnancy, the pregnant pigs were euthanised for sample collection, according to the AEC approved protocol. The gravid uterus was harvested by midventral laparotomy. Then, the uterus was opened via an incision along the anti-mesometrial side of the uterine horn. In each uterus, one uterine horn was randomly identified, from which three focal fetuses were collected (one each from the cervical, middle, and tip uterine positions). Their matched placentae were identified and carefully stripped from the endometrium and weighted. Fetal and placental weights were measured, and placental efficiency (fetal/placental weight) was calculated. Placentae were placed flat on cutting mats and the surface areas were measured. A placental biopsy sample was collected and immediately snap frozen in liquid nitrogen and stored at −80 °C prior to further analysis. 

### 5.4. Total RNA Isolation, Library Preparation, and Sequencing

A total of 10 female placentae (CON, *n* = 5 placentae; HS, *n* = 5 placentae) from 10 pregnant pigs were selected for the RNA-seq experiment. The selection criteria were to collect (1) a representative sample from each litter in consideration of within-litter variation in litter-bearing species [41]; (2) a representative sample with placental efficiency (fetal/placental weight) being closest to the average placental efficiency (fetal/placental weight) among the focal litter, and (3) exclusively female placentae, as they exhibited a greater reduction in placental efficiency to maternal heat exposure compared to their male counterparts from our previously experimental data [15]. Placental total RNA was extracted using ReliaPrep RNA tissue Miniprep System (Cat# Z6111; Promega, Madison, WI, USA) as per the manufacturer’s instructions. RNA integrity and concentration were assessed using the RNA Nano 6000 Assay Kit of the Bioanalyzer 2100 system (Agilent Technologies, Santa Clara, CA, USA) according to the manufacturer’s instructions. RNA purity was assessed using a nanodrop spectrophotometer (Thermo Scientific, Walton, MA, USA). The placental total RNA for sequencing library preparation had an average 28S/18S ratio of 1.3 ± 0.2 (SD) and an average RNA integrity number (RIN) of 8.4 ± 0.3 (SD). The sequencing library was constructed using the NEBNext^®^ Ultra™ Directional RNA Library Prep Kit for Illumina^®^ (New England Biolabs, Ipswich, MA, USA) according to the manufacturer’s instructions. The library was sequenced on an Illumina NovaSeq6000 sequencing platform (Illumina, Inc., San Diego, CA, USA), and 150 bp paired-end reads were generated from each placental sample. The raw sequencing data used for the analysis were deposited in the NCBI’s gene expression omnibus public repository [42] with the accession number GSE168571 (https://www.ncbi.nlm.nih.gov/geo/query/acc.cgi?acc=GSE168571, accessed on 9 March 2021). 

### 5.5. Sequencing Data Reprocessing 

Quality control of sequencing reads was performed using FastQC software version 0.11.9 (http://www.bioinformatics.babraham.ac.uk/projects/fastqc, accessed on 18 March 2020). The raw reads were subjected to Illumina adaptor and low-quality base removals to obtain clean reads. The clean reads for downstream reprocessing and analyses had a Phred score above 30 equivalent to a nucleobase accuracy above 99.9%. The pig reference genome (*Sus scrofa* 11.1) DNA sequence (FASTA) and annotation GTF files were obtained from Ensembl (https://www.ensembl.org/Sus_scrofa/Info/Index, accessed on 26 March 2020). Clean reads were mapped to the pig reference genome (*Sus scrofa* 11.1) using Hisat2 version 2.1.0 [43]. The average alignment rate across all samples was 96.5% ± 0.15 (SD). The aligned reads were then counted per gene using HTSeq software (version 0.11.3) with reverse strand interpretation [44].

### 5.6. Differential Gene Expression, and Gene Ontology (Go) and Pathway Enrichment Analyses

Differentially expressed genes (DEGs) between placentae from CON and HS groups were analysed using DEseq2 package version 1.28.1 [45] in R version 4.0.0 [46]. To control false positive findings, raw *p* values were subjected to the Benjamini-Hochberg (BH) false discovery rate (FDR) correction for multiple testings. Genes with an adjusted *p* value (FDR) ≤ 0.1 were considered as differentially expressed [36]. Candidate DEGs were then subjected to gene ontology (GO; domains of molecular function, biological process and cellular component) and pathway enrichment analyses (statistical overrepresentation test) using the PANTHER dataset version 16.0 [47], annotated for the *Sus scrofa* database. The pathway analyses were also performed using g:Profiler [48]. Upregulated and downregulated candidate DEGs (HS compared with CON) were analysed separately. Gene ontology and pathway terms with an adjusted *p* value (FDR) ≤ 0.05 were considered enriched or overrepresented. 

### 5.7. Western Blot Analysis

Among the nutrient transporters encoded by the DEGs, two proteins, PEPT1 (*SLC15A1*) and GLUT2 (*SLC2A2*), were chosen to further identify relative protein expression by western blot analysis, as the antibodies for the pig are available. Placental total proteins (CON, *n* = 5 placentae; HS, *n* = 5 placentae) were extracted using RIPA lysis and extraction buffer (Cat# 89900, ThermoFisher Scientific, Waltham, MA, USA), as per the manufacturer’s instructions. Total protein concentrations were measured using the Pierce BCA protein assay kit (Cat#23225, ThermoFisher Scientific, MA, USA). Identical total protein amounts from each sample were loaded onto the NuPAGE Tris-Acetate Mini Protein Gel (Catalog # EA03752BOX, Invitrogen, Carlsbad, CA, USA) and separated at 150 volts (V) for 55 min. The separated proteins were electrophoretically transferred to a nitrocellulose membrane using iBlot Dry Blotting System (ThermoFisher Scientific, Waltham, MA, USA), as per the manufacturer’s instructions. The membranes were stained using the Revert 700 total protein stain and wash solution kit (Cat# 926-11015, LI-COR Biosciences, Lincoln, NE, USA), as per the manufacturer’s instructions, followed by the total protein visualisation at 700 nm channel using the Odyssey^®^ FC Imaging System (LI-COR Biosciences, Lincoln, NE, USA). The membranes were blocked in 5% non-fat dry milk in mixture of tris-buffered saline (TBS) and Tween 20 (TBST) for 2 h at room temperatures under gentle agitation. The membranes were then incubated with anti-*SLC2A2* rabbit (at 1 µg/mL, Cat# ARP41706_P050, AVIVA Systems Biology, San Diego, CA, USA) and anti-*SLC15A1* mouse (1:500 dilution, Cat# sc-373742, Santa Cruz Biotechnology, Dallas, TX, USA) primary antibodies diluted in TBST containing 5% non-fat dry milk overnight at 4 °C. The blots were extensively washed with TBST buffer and incubated with Donkey anti-Rabbit (1:15,000 dilution, Cat# 926-32213, LI-COR Biosciences, NE, USA) and Donkey anti-Mouse (1:15,000 dilution, Cat# 926-32212, LI-COR Biosciences, NE, USA) IgG secondary antibodies diluted in TBST containing 5% non-fat dry milk for 1 h at room temperatures under gentle agitation. Fluorescence bands were visualised at 800 nm channel with the Odyssey^®^ FC Imaging System (LI-COR Biosciences, NE, USA). The images were analysed using Empiria Studio (LI-COR Biosciences, NE, USA). Relative protein expression of each sample was normalised to total protein and expressed as normalised signals.

### 5.8. Statistics for Placental and Fetal Morphology, and Western Blot

Data normality was verified. Placental and fetal morphological parameters, and normalised signals generated from western blot images between control and heat stress groups were analysed using Student’s t-test. A *p* value ≤ 0.05 was considered significantly different. 

## Figures and Tables

**Table 1:** Placental and fetal morphology from pregnant pigs exposed to thermoneutral control (CON, constant 20 °C; *n* = 5) or cyclic heat stress (HS, 28 to 33 °C; *n* = 5) conditions from d40 to d60 of gestation.

Variables	Treatments		*p* Values
	CON (*n* = 5)	HS (*n* = 5)	
Placental weight (g)	88 ± 6	112 ± 12	0.053
Fetal weight (g)	104 ± 7	92 ± 4	0.08
Placental efficiency (fetal/placental weight)	1.20 ± 0.09	0.87 ± 0.11	0.023
Placental surface area (cm^2^)	725 ± 59	824 ± 31	0.09
Data are expressed as means with standard error of the means (SEM).			

**Table 2:** A subset of differentially expressed genes in heat stress placentae (*n* = 5) compared to control placentae (*n* = 5).

Gene Symbol	Full Gene Name	Log2 Fold Change	False Discovery Rate
*IGSF5*	immunoglobulin superfamily member 5	2.04	7 × 10^−6^
*ZNF691*	zinc finger protein 691	0.70	9 × 10^−4^
*SLC7A10*	solute carrier family 7 member 10	0.64	4 × 10^−3^
*ABTB1*	ankyrin repeat and BTB domain containing 1	0.53	7 × 10^−3^
*MRVI1*	murine retrovirus integration site 1 homolog	0.45	7 × 10^−5^
*OSGIN1*	oxidative stress induced growth inhibitor 1	0.39	2 × 10^−2^
*SLC30A2*	solute carrier family 30 member 2	0.34	6 × 10^−2^
*SLC26A6*	solute carrier family 26 member 6	0.28	3 × 10^−2^
*SLC25A25*	solute carrier family 25 member 25	0.19	7 × 10^−2^
*GPI*	glucose-6-phosphate isomerase	−0.16	7 × 10^−2^
*ATP1A1*	ATPase Na+/K+ transporting subunit alpha 1	−0.19	8 × 10^−2^
*GPRC5C*	G protein-coupled receptor class C group 5 member C	−0.22	5 × 10^−2^
*SLC11A2*	solute carrier family 11 member 2	−0.24	9 × 10^−2^
*SLC8B1*	solute carrier family 8 member B1	−0.24	8 × 10^−2^
*SCNN1G*	sodium channel epithelial 1 gamma subunit	−0.26	6 × 10^−2^
*SLC20A1*	solute carrier family 20 member 1	−0.28	4× 10^−2^
*SLC40A1*	solute carrier family 40 member 1	−0.32	2 × 10^−2^
*ATP8B1*	ATPase phospholipid transporting 8B1	−0.32	4 × 10^−2^
*DEFB1*	defensin beta 1	−0.41	7 × 10^−2^
*IGFBP5*	insulin-like growth factor-binding protein 5	−0.45	6 × 10^−2^
*ATP13A3*	ATPase 13A3	-0.60	1 × 10^−2^
*ST3GAL1*	ST3 beta-galactoside alpha-2,3-sialyltransferase 1	−0.60	2 × 10^−3^
*SLC2A2*	solute carrier family 2 member 2	−0.62	1 × 10^−2^
*B3GNT7*	betaGal beta-1,3-N-acetylglucosaminyltransferase 7	−0.73	6 × 10^−7^
*ST3GAL3*	ST3 beta-galactoside alpha-2,3-sialyltransferase 3	−0.75	1 × 10^−3^
*SLC38A3*	solute carrier family 38 member 3	−0.76	2 × 10^−2^
*FABP5*	fatty acid binding protein 5	−0.79	8 × 10^−4^
*ANXA1*	annexin A1	−0.81	8× 10^−4^
*SLC7A8*	solute carrier family 7 member 8	−0.87	7 × 10^−4^
*CCN2*	cellular communication network factor 2	−0.88	5 × 10^−4^
*ECM1*	extracellular matrix protein 1	−1.00	2 × 10^−4^
*PTGER4*	prostaglandin E receptor 4	−1.15	2 × 10^−3^
*KCNK5*	potassium two pore domain channel subfamily K member 5	−1.20	1 × 10^−5^
*SLC45A3*	solute carrier family 45 member 3	−1.37	6 × 10^−8^
*FBP2*	fructose-bisphosphatase 2	−1.44	5 × 10^−4^
*SPINK4*	serine peptidase inhibitor, Kazal type 4	−1.45	1× 10^−8^
*SLC15A1*	solute carrier family 15 member 1	−1.46	1 × 10^−13^
*IRX3*	iroquois homeobox 3	−1.50	3 × 10^−12^
*LHFPL4*	LHFPL tetraspan subfamily member 4	−1.60	1 × 10^−6^
*CHST8*	carbohydrate sulfotransferase 8	−1.75	3 × 10^−7^

**Table 3:** Pathways significantly enriched by the downregulated genes in heat stress placentae (*n* = 5) compared with control placentae (*n* = 5).

Pathway Database	Term	Pathway ID	FDR (False Discovery Rate)	Number of Genes Enriched
Reactome	Lysosomal oligosaccharide catabolism	R-SSC-8853383	0.042	2
	* Metabolism of carbohydrates	R-SSC-71387	0.000	10
	Keratan sulfate biosynthesis	R-SSC-2022854	0.007	4
	* Glycosaminoglycan metabolism	R-SSC-1630316	0.008	6
	Sialic acid metabolism	R-SSC-4085001	0.009	4
	* Synthesis of substrates in N-glycan biosynthesis	R-SSC-446219	0.043	4
	O-linked glycosylation of mucins	R-SSC-913709	0.043	4
	* O-linked glycosylation	R-SSC-5173105	0.044	5
	Transport of inorganic cations/anions and amino acids/oligopeptides	R-SSC-425393	0.039	5
	* SLC-mediated transmembrane transport	R-SSC-425407	0.008	9
KEGG	Metabolic pathways	KEGG:01100	0.001	31
	Lysosome	KEGG:04142	0.002	8
	Glycosaminoglycan biosynthesis—keratan sulfate	KEGG:00533	0.027	3
	Glycosphingolipid biosynthesis—ganglio series	KEGG:00604	0.034	3

* Where multiple pathway terms with loose hierarchy are significantly enriched, a broad ‘parent’ term and an associated ‘child’ term above are listed. KEGG: Kyoto Encyclopedia of Genes and Genomes.

## Data Availability

The raw sequencing data used for the analysis were deposited in the NCBI’s gene expression omnibus public repository with the accession number GSE168571 (https://www.ncbi.nlm.nih.gov/geo/query/acc.cgi?acc=GSE168571, accessed on 9 March 2021).

## Supplementary Materials

The following are available online at https://www.mdpi.com/article/10.3390/ijms22084147/s1, Table S1: Gene ontology enrichment analysis of downregulated genes (HS compared with CON). Table S2: Pathway enrichment analysis of downregulated genes (HS compared with CON). Supporting Information File 1: Original western blot images for GLUT2 and PEPT1.
